# Supplementary material for: Setting the photoelectron clock through molecular alignment
Source: Nat Commun. 2020 May 21;11:2546. doi: 10.1038/s41467-020-16270-0 (PMC7242449; doi:10.1038/s41467-020-16270-0)
Supplement: Supplementary file 1 — Supplementary Information [file 41467_2020_16270_MOESM1_ESM.pdf]

Supplementary information:
Setting the photoelectron clock through molecular alignment

Trabattoni et al.

SUPPLEMENTARY NOTE 1: NUMERICAL QUANTUM-DYNAMICS SIMULATIONS

Numerical simulations of the full LIED dynamics have been performed from first-principles within the time-dependent density functional theory (TDDFT) [1] framework as implemented in the real-space real-time Octopus code [2]. In TDDFT, the dynamics of an interacting many-electron system is cast into the manageable problem of a fictitious non-interacting system under the effect of a time-dependent potential such that the non-interacting and the interacting systems have the same time-dependent density.

Since core electrons are expected to play a marginal role in the experiment we consider only valence electrons and account for inner-shell electrons by the effect of norm-conserving Troullier-Martins pseudopotentials. To obtain a good description of ionisation, we employed a local density approximation (LDA) functional with the average density self-interaction correction (ADSIC) [3], which corrects the asymptotic decay and provides a first and second ionisation energy of 11.65 eV and 15.69 eV, in good agreement with experimental values [4]. During the simulations the nuclei are held fixed in the equilibrium positions, $r_{\mathrm{C–S}} = 156.1$ pm and $r_{\mathrm{C–O}} = 115.6$ pm.

The TDDFT equations are discretised in real-space with a cartesian grid of spacing 0.4 a. u. with a cylindrical shape of radius 50 a. u. and length 260 a. u. aligned along the laser-polarisation direction. The solution of the electron dynamics is obtained by using a discretised real-time evolution with a time step of 0.08 a. u.. The calculations are performed with a 30 fs laser pulse. Complex absorbing boundaries of varying thicknesses, 40 a. u. from the caps of the cylinder and 10 a. u. on the radial borders, are placed at the edges of the simulation box to prevent spurious reflections [5].

The photoelectron spectrum is calculated by collecting the flux of the photoionisation current through a spherical surface of radius 40 a. u. with the tSURFF method [6, 7]. This approach gives access to the momentum resolved photoelectron probability $I(\mathbf{p})$ from which, by integrating along the direction perpendicular to the detector, it is possible to obtain the angular distribution of the experiment: $I(p_X, p_Y) = \int \mathrm{d}p_Z\, I(\mathbf{p})$.

The non-perfect molecular alignment in the laboratory frame is accounted for by sampling the relative angle θ between the laser polarisation and the molecular axis from $0\,^\circ$ to $90\,^\circ$ in steps of $10\,^\circ$, as shown in Supplementary Figure 1. This procedure requires a separate simulation for each θ. The photoelectron spectra for a given configuration, parallel or perpendicular, are obtained by averaging the photoelectron distributions $I_\theta(\mathbf{p})$ with weights $n_\theta(\theta - \theta^{\parallel/\perp}) = \exp(-\sin(\theta - \theta^{\parallel/\perp})^2/(2\sigma^2))$, $\sigma^2 = 1 - \langle \cos^2\theta_{\mathrm{2D}} \rangle$, and $\langle \cos^2\theta_{\mathrm{2D}} \rangle = 0.9$.

Furthermore, to account for the rotation of the molecule about the polarisation axis for parallel alignment we impose cylindrical symmetry of the photoelectron distribution about Y by averaging over ϕ: $\bar{I}_\phi(\mathbf{p}) = (2\pi)^{-1} \int_0^{2\pi} \mathrm{d}\phi\, R_\phi\left(I(\mathbf{p})\right)$ with the operator R_ϕ of rotation in the X, Z plane. The final spectrum is obtained as follows:

$$\bar{I}^{\parallel/\perp}(p_X, p_Y) = \int \mathrm{d}p_z \int \mathrm{d}\theta\, n(\theta - \theta^{\parallel/\perp}) \bar{I}_{\phi,\theta}(\mathbf{p})\,. \quad (1)$$

To account for experimental-background in the simulations, a constant offset of 2×10^{-8} was added to the energy distributions, see Supplementary Figure 2. For comparison, the spectra for perfectly aligned configurations are reported in Supplementary Figure 3. We point out that the background correction shifts the numerically obtained cutoffs to lower energy, but does not affect the general behaviour nor the difference of the cutoffs between the parallel and perpendicular configurations.

From the numerical simulations the crucial role of the usually neglected electron-electron interaction for correctly describing the cutoff region in the parallel configuration became evident. Supplementary Figure 4 a shows the decomposed contributions of the Kohn-Sham HOMO and HOMO-1 orbitals, which highlight their distinct contributions to two distinct cutoffs, which are strongly separated in intensity. In particular, the faint 10 U_p cutoff for the parallel case actually appears to be uniquely determined by the HOMO-1, which does not have a node along the molecular axis, whereas contributions from the HOMO were strongly suppressed by the presence of a node along the molecular axis, i. e., parallel to the laser-polarisation axis. Second, the independent particle simulation obtained by propagating the system with the Hartree, exchange, and correlation potentials frozen, mimicking the widely used single-active electron model, presents a qualitatively different picture, see Supplementary Figure 4 b. In particular the contribution of the HOMO-1 is highly overestimated and for the parallel alignment the 10 U_p cutoff is restored, in clear contradiction with the experiment. These results also confirm the importance of the coherent interaction between different orbitals in strong-field ionisation [8].

SUPPLEMENTARY NOTE 2: SEMICLASSICAL TRAJECTORY SIMULATIONS

Semiclassical trajectory simulations were carried out employing a simplified MO-ADK (molecular-orbital Ammosov-Delone-Kraĭnov) approach, to create the initial wavepacket, in conjunction with a classical continuum propagation in the combined laser-electric and Coulomb field of the cation.

Initial electron wavepacket

In general, the phase-space distribution of the initial electron wavepacket was created in a similar fashion as described before [9]. The ionisation probability in dependence of the instantaneous electric field was obtained through the quasistatic ADK tunnelling theory supplemented by an empirical extension to the barrier-suppression regime [10]. Electric-field dependent ionisation potentials, $I_p(\vec{\epsilon})$, were computed through second-order perturbation theory

$$I_p(\vec{\epsilon}) = I_p^{(0)} - \Delta\vec{\mu}\cdot\vec{\epsilon} - \frac{1}{2}\vec{\epsilon}^{\,\mathrm{T}}\Delta\alpha\,\vec{\epsilon}, \tag{2}$$

assuming that ionisation occurs exclusively from the HOMO, as supported by the TDDFT results. $I_p^{(0)}$ is the field-free ionisation potential, $\Delta\vec{\mu}$ and $\Delta\alpha$ are the differences of dipole moment and polarizability tensor between cationic and neutral species, respectively. Here, the measured field-free ionisation potential of $I_p^{(0)} = 11.19$ eV [4] was used in combination with calculated neutral and cationic dipole moments and polarizabilities [9]. In Supplementary Figure 5 the resulting time-of-birth distribution of a typical electron wavepacket is shown.

The classical tunnel exit was composed as $\vec{r}_0 = -\vec{\epsilon}\,I_p/\epsilon^2$ [11]. The initial momentum distribution in the plane transverse to the electric field vector at the instance of ionisation was modelled according to the atomic ADK tunnelling theory with additional imprint of the initial electronic state's nodal structure. The initial momentum component along the electric field vector was obtained through nonadiabatic tunnelling theory [12]. For the parallel-alignment case the nodal line of the HOMOs along the molecular axis imprints onto the momentum distribution at birth. Accordingly, the initial transverse momentum distribution for the parallel alignment case was described as

$$\omega_{\parallel}\left(p_{0,x}, p_{0,z}\right) \propto \left(p_{0,x}^2 + p_{0,z}^2\right) \cdot \mathrm{e}^{-\frac{\sqrt{2I_p}}{\epsilon}\left(p_{0,x}^2 + p_{0,z}^2\right)} . \tag{3}$$

For the perpendicular alignment case the nodal plane orthogonal to the molecular axis imprints onto the initial momentum distribution. Since it splits the HOMO's electron density unequally with a ratio of 85:15 [9], its imprint was described as a nodal plane with a damped peculiarity

$$\beta = 1 - \frac{|\varrho_+ - \varrho_-|}{\varrho_+ + \varrho_-} , \tag{4}$$

with $\varrho_\pm$ representing the integral electron densities on the two sides of the nodal plane. Hence, the distribution of initial transverse momenta for the perpendicular alignment case was set up as

$$\omega_{\perp}\left(p_{0,x}, p_{0,z}\right) \propto \left|p_{0,z}\right|^{2\beta} \cdot \mathrm{e}^{-\frac{\sqrt{2I_p}}{\epsilon}\left(p_{0,x}^2 + p_{0,z}^2\right)} . \tag{5}$$

Supplementary Figure 6 illustrates exemplary transverse momentum distributions for both alignment cases.

Classical propagation

The electron wavepacket at birth into the continuum was sampled from the initial phase-space distribution through rejection sampling, expanded as a coherent superposition of partial plane waves and, subsequently, propagated in the combined electric laser and the cation's Coulomb field. The singly charged cation was represented by a point charge $+e$.

The asymptotic electron phase after exposure to the combined electric field of laser and point charge was obtained through [13]

$$\phi_\infty = -\vec{p}_0\cdot\vec{r}_0 + I_p\cdot t_0 - \int_{t_0}^{\infty}\left(\frac{p^2}{2} - \frac{2}{r}\right)\mathrm{d}t\,. \tag{6}$$

At short distances between the electron and the point charge, $r < r_{2b}$, the laser-electric field becomes negligible and the description of the electron motion reduces to a two-body problem. Here, the threshold distance, r_{2b}, was chosen such that the corresponding Coulomb field is 1000 times larger than the peak electric field of the laser. Accordingly, for the experimental peak electric field of $\epsilon_0 \approx 0.048$ a. u. this threshold distance becomes $r_{2b} = 1/\sqrt{1000\,\epsilon_0} \approx 0.14$ a. u.. The motion of the electron within the spherical volume of radius r_{2b} around the point charge could then be described conveniently as a Kepler orbit. This approximation allows for direct computation of the electron's properties at its symmetric exit point from the sphere: Its position and momentum vector at exit as well as its time of flight between entry and exit of this sphere can be computed fully analytically. The phase accumulated during its passage through the sphere is accessible through low-effort numerical integration. In Supplementary Figure 7 a typical trajectory of an electron is shown as it performs a swing-by around the point charge.

SUPPLEMENTARY REFERENCES

[1] M. A. L. Marques, N. T. Maitra, F. Nogueira, E. K. U. Gross, and A. Rubio, *Fundamentals of Time-Dependent Density Functional Theory*, Lecture Notes in Physics (Springer Verlag, 2011).

[2] X. Andrade, D. Strubbe, U. De Giovannini, A. H. Larsen, M. J. T. Oliveira, J. Alberdi-Rodriguez, A. Varas, I. Theophilou, N. Helbig, M. J. Verstraete, L. Stella, F. Nogueira, A. Aspuru-Guzik, A. Castro, M. A. L. Marques, and A. Rubio, Real-space grids and the octopus code as tools for the development of new simulation approaches for electronic systems, Phys. Chem. Chem. Phys. **17**, 31371 (2015).

[3] C. Legrand, E. Suraud, and P. G. Reinhard, Comparison of self-interaction-corrections for metal clusters, J. Phys. B-Atom. Mol. Opt. **35**, 1115 (2002).

[4] L. S. Wang, J. E. Reutt, Y. T. Lee, and D. A. Shirley, High-resolution UV photoelectron-spectroscopy of CO_2^+, COS^+ and CS_2^+ using supersonic molecular-beams, J. Electron. Spectrosc. Relat. Phenom. **47**, 167 (1988).

[5] U. De Giovannini, A. H. Larsen, and A. Rubio, Modeling electron dynamics coupled to continuum states in finite volumes with absorbing boundaries, Eur. Phys. J. D **88**, 1 (2015).

[6] L. Tao and A. Scrinzi, Photo-electron momentum spectra from minimal volumes: the time-dependent surface flux method, New J. Phys. **14**, 013021 (2012).

[7] P. Wopperer, U. De Giovannini, and A. Rubio, Efficient and accurate modeling of electron photoemission in nanostructures with TDDFT, Eur. Phys. J. D **90**, 1307 (2017).

[8] H. Akagi, T. Otobe, A. Staudte, A. Shiner, F. Turner, R. Dörner, D. Villeneuve, and P. Corkum, Laser tunnel ionization from multiple orbitals in HCl, Science **325**, 1364 (2009).

[9] L. Holmegaard, J. L. Hansen, L. Kalhøj, S. L. Kragh, H. Stapelfeldt, F. Filsinger, J. Küpper, G. Meijer, D. Dimitrovski, M. Abu-samha, C. P. J. Martiny, and L. B. Madsen, Photoelectron angular distributions from strong-field ionization of oriented molecules, Nat. Phys. **6**, 428 (2010), arXiv:1003.4634 [physics].

[10] X. M. Tong and C. D. Lin, Empirical formula for static field ionization rates of atoms and molecules by lasers in the barrier-suppression regime, J. Phys. B **38**, 2593 (2005).

[11] X.-B. Bian, Y. Huismans, O. Smirnova, K.-J. Yuan, M. J. J. Vrakking, and A. D. Bandrauk, Subcycle interference dynamics of time-resolved photoelectron holography with midinfrared laser pulses, Phys. Rev. A **84**, 043420 (2011).

[12] M. Li, J.-W. Geng, M. Han, M. M. Liu, L.-Y. Peng, Q. Gong, and Y. Liu, Subcycle nonadiabatic strong-field tunneling ionization, Phys. Rev. A **93**, 013402 (2016).

[13] N. I. Shvetsov-Shilovski, M. Lein, L. B. Madsen, E. Räsänen, C. Lemell, J. Burgdörfer, D. G. Arbó, and K. Tőkési, Semiclassical two-step model for strong-field ionization, Phys. Rev. A **94**, 013415 (2016).

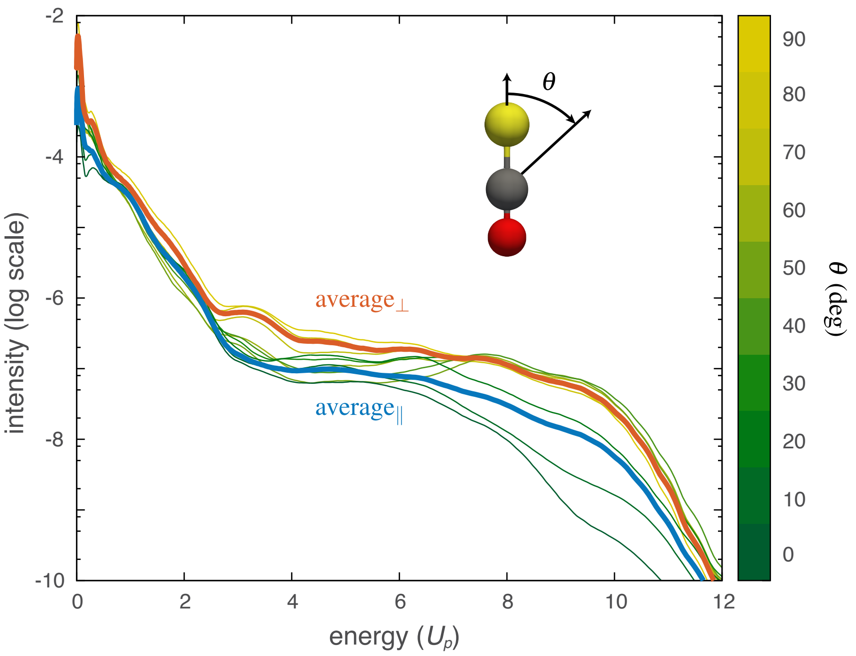

Supplementary Figure 1. Simulated photoelectron spectra as a function of the angle θ between the laser polarisation and the molecular axis. The averaged spectra for a parallel and perpendicular alignment with a degree of alignment of $\left\langle \cos^2\theta_{2D} \right\rangle = 0.9$ are reported with thick lines.

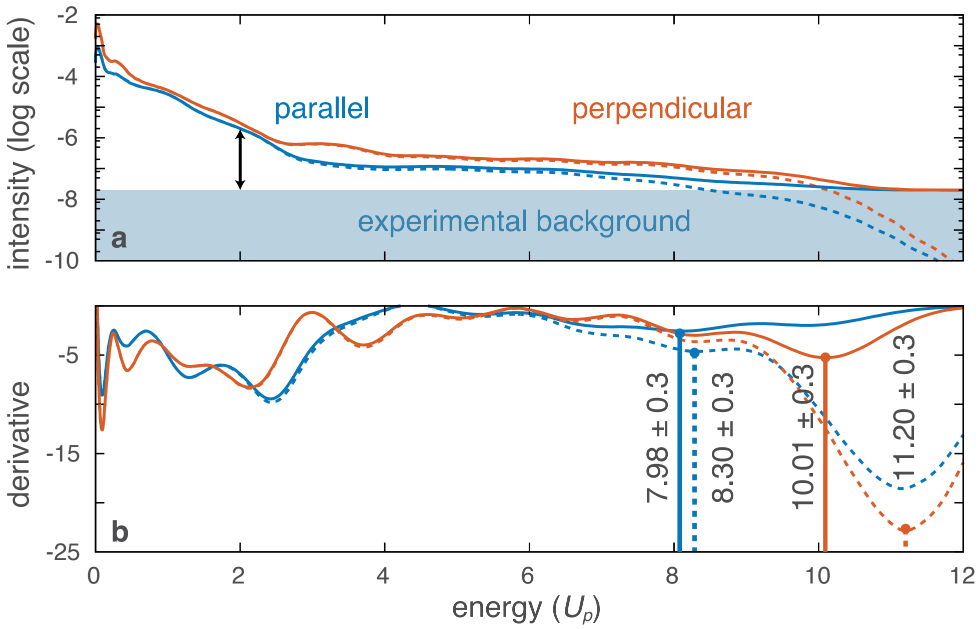

Supplementary Figure 2. Effect of the experimental-background in the simulated photoelectron spectra. **a**: Simulated photoelectron distributions, for parallel and perpendicular alignment, averaged over θ according to the degree of alignment in the experiment $\bar{I}_{\parallel/\perp}(E)$ (dashed lines). A constant, 2×10^{-8}, is added to the simulated data to account for the experimental-background level (solid lines), i. e., to match the experimental contrast from 2 to 12 U_p. **b**: Derivatives of the photoelectron distributions. Regardless of the presence of the background the cutoffs for the parallel and perpendicular configurations present an observable difference which is in qualitative agreement with the results of Fig.2 of the main manuscript.

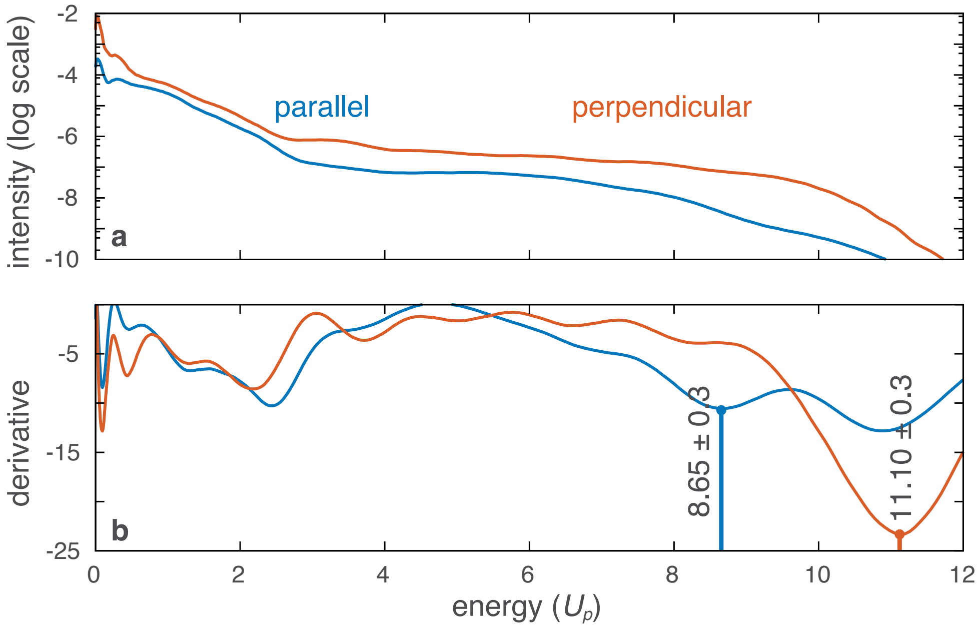

Supplementary Figure 3. Same as Supplementary Figure 2 for perfect alignment and no experimental-background correction.

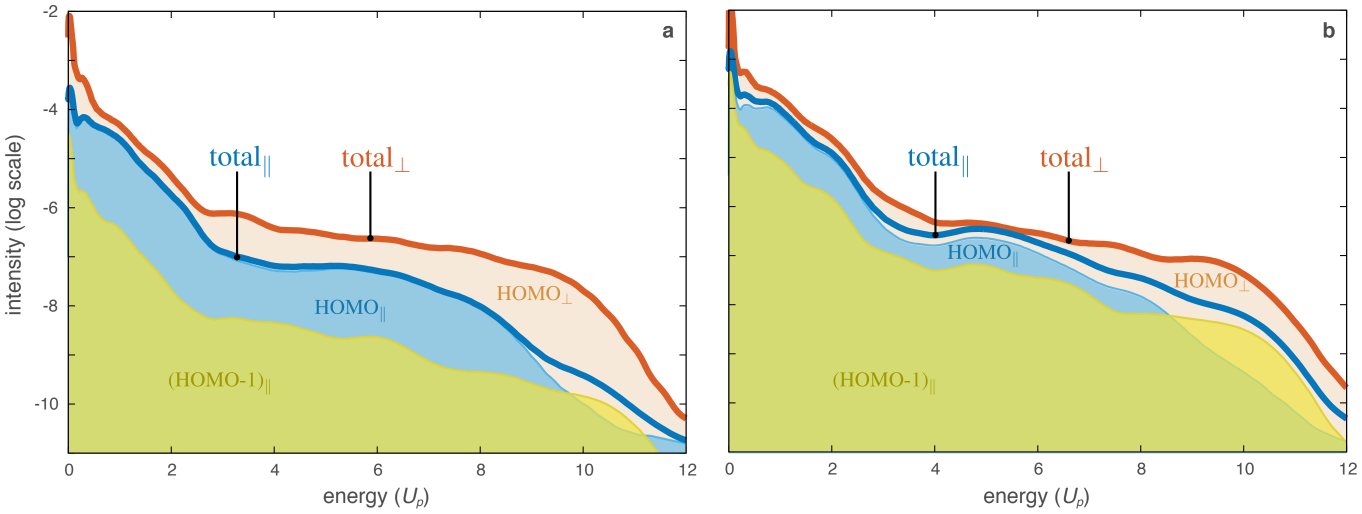

Supplementary Figure 4. Role of electron-electron interaction and orbital contribution to the total photoelectron spectrum for perfect alignment. **a**: Spectra obtained with the fully-interacting-electrons TDDFT equations. **b**: Spectra for the non-interacting-electrons simulation obtained by freezing the Hartree exchange and correlation potentials to the neutral ground state potentials.

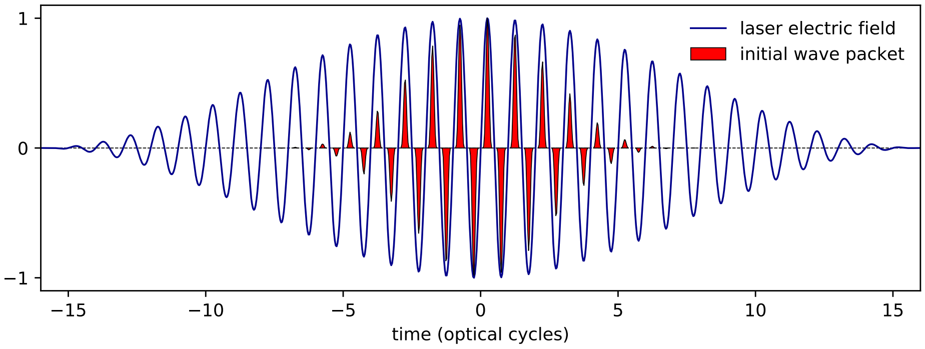

Supplementary Figure 5. Time-of-birth distribution of an electron wavepacket for OCS at perpendicular alignment as obtained from the quasistatic ADK tunnelling theory. The distribution shown was normalised with respect to its maximum and multiplied with the sign of the instantaneous electric field. The electric field is displayed in units of ϵ_0.

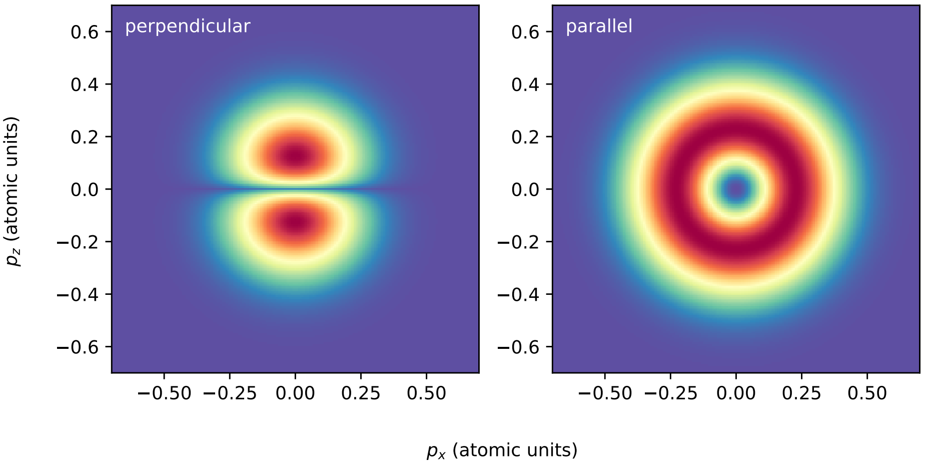

Supplementary Figure 6. Transverse initial momentum distributions assuming the field-free ionisation potential and peak electric field.

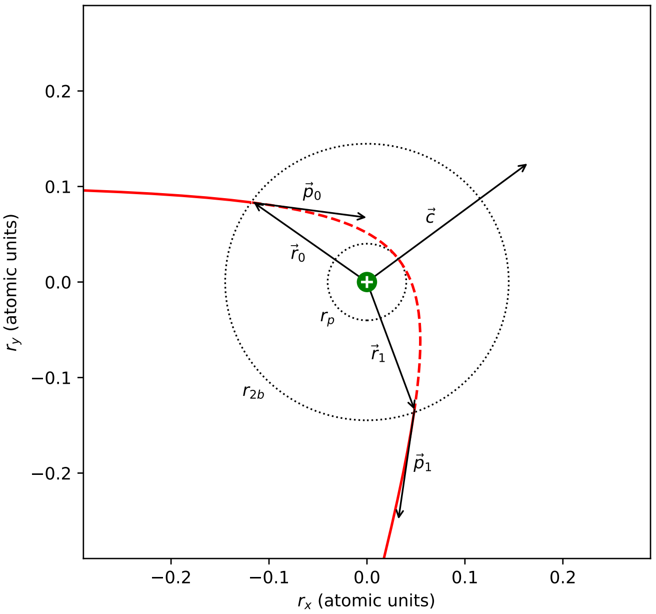

Supplementary Figure 7. Example trajectory in the combined laser-electric and Coulomb field of a point charge $+e$ (green dot), numerically propagated for $r > r_{2b}$ (solid red line) and analytically approximated for $r \leq r_{2b}$ by means of a pure two-body interaction (dashed red line). By deduction of the orbital centre $\vec{c}$ and the distance at closest approach, the periapsis r_p, the symmetric exit position $\vec{r}_1$, the corresponding momentum vector $\vec{p}_1$, and the time spent within the sphere can be obtained analytically.
